# Degradation of PET Bottles by an Engineered *Ideonella sakaiensis* PETase

**DOI:** 10.3390/polym15071779

**Published:** 2023-04-03

**Authors:** Maria Eduarda Sevilla, Mario D. Garcia, Yunierkis Perez-Castillo, Vinicio Armijos-Jaramillo, Santiago Casado, Karla Vizuete, Alexis Debut, Liliana Cerda-Mejía

**Affiliations:** 1 Facultad de Ciencia e Ingeniería en Alimentos y Biotecnología, Universidad Técnica de Ambato, Ambato 180216, Ecuador; 2 Área de Ciencias Aplicadas, Facultad de Ingeniería y Ciencias Aplicadas, Universidad de Las Américas, Quito 170125, Ecuador; 3 Grupo de Bio-Quimioinformática, Universidad de Las Américas, Quito 170125, Ecuador; 4 Ingeniería en Biotecnología, Facultad de Ingeniería y Ciencias Aplicadas, Universidad de Las Américas, Quito 170125, Ecuador; 5 Centro de Nanociencia y Nanotecnología, Universidad de Las Fuerzas Armadas ESPE, Sangolquí 171103, Ecuador; 6 Departamento de Ciencias de la Vida y Agricultura, Universidad de Las Fuerzas Armadas ESPE, Sangolquí 171103, Ecuador

**Keywords:** *Is*PETase, biodegradation, polyethylene terephthalate, PET, enzyme engineering

## Abstract

Extensive plastic production has become a serious environmental and health problem due to the lack of efficient treatment of plastic waste. Polyethylene terephthalate (PET) is one of the most used polymers and is accumulating in landfills or elsewhere in nature at alarming rates. In recent years, enzymatic degradation of PET by *Ideonella sakaiensis* PETase (*Is*PETase), a cutinase-like enzyme, has emerged as a promising strategy to completely depolymerize this polymer into its building blocks. Here, inspired by the architecture of cutinases and lipases homologous to *Is*PETase and using 3D structure information of the enzyme, we rationally designed three mutations in *Is*PETase active site for enhancing its PET-degrading activity. In particular, the S238Y mutant, located nearby the catalytic triad, showed a degradation activity increased by 3.3-fold in comparison to the wild-type enzyme. Importantly, this structural modification favoured the function of the enzyme in breaking down highly crystallized (~31%) PET, which is found in commercial soft drink bottles. In addition, microscopical analysis of enzyme-treated PET samples showed that *Is*PETase acts better when the smooth surface of highly crystalline PET is altered by mechanical stress. These results represent important progress in the accomplishment of a sustainable and complete degradation of PET pollution.

## 1. Introduction

Plastics have become a crucial component of modern society due to their exceptional mechanical and physical properties, including durability, flexibility, and impermeability [1]. The demand for plastics led to the production of 367 Mt in 2020 [2]. However, only 9% of plastic waste generated undergoes conventional recycling processes, with the rest ending up in landfills or elsewhere in the environment [3]. The continuing accumulation of plastic raises significant concerns about its impact on human and wildlife health. While approximately 100,000 marine animals are killed every year by entanglement or ingestion of macroplastics (>5 mm) [4], recent studies suggest that microplastic (1 µm–5 mm) contamination may reduce the survival of fish and cause oxidative stress and neurotoxic effects that lead to behavioural disorders [5,6]. In humans, plastic pollution and its by-products induce negative sequels in different organs, including the lungs, skin, liver, brain and gastrointestinal system, which triggers an immune response, cell toxicity, cancer and neurological disorders, along with other health issues [7].

**Figure 1:**
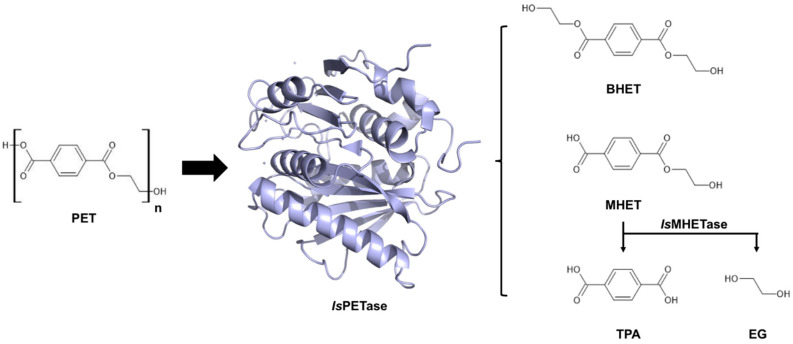
Chemical structure of *Is*PETase substrate and products. PET: polyethylene terephthalate, BHET: bis(2-hydroxyethyl) terephthalic acid, MHET: mono(2-hydroxyethyl) terephthalic acid, TPA: terephthalic acid, EG: ethylene glycol.

Polyethylene terephthalate (PET) (Figure 1), obtained by polycondensation of terephthalic acid (TPA) and ethylene glycol (EG) or the transesterification of dimethyl terephthalate (DMT) and EG [8], is considered the most abundant thermoplastic material. Its low price and remarkable durability and impermeability to liquids and gases make it ideal for the fabrication of single-use soft drink bottles and packing [2]. Due to these favourable properties, PET is highly resistant to biodegradation and accumulates in nature. New materials with higher biodegradability, such as polylactic acid (PLA), polyhydroxyalkanoate (PHA), and polybutylene succinate (PBS), have emerged as a measure to reduce the environmental impact of PET [1]. However, certain applications in the food industry require the use of packing materials exclusively made of PET [9] or using virgin PET [10] to comply with safety regulations, which sustains PET consumption. This scenario highlights the need for new strategies to recycle PET sustainably.

PET is mostly recycled through thermal treatments, but these processes cause degradation of the polymer structure and loss of some mechanical properties required for beverage bottles [11]. Alternatively, PET is depolymerised by pyrolysis or gasification to yield fuels used to produce heat and electricity. These processes are inexpensive and generate a high profit margin, but they have significant environmental drawbacks, such as heavy pollution and a contribution to the build-up of greenhouse gases [12]. Moreover, PET can be chemically broken down into monomers using solvents, such as alcohol, and a catalyst. The resulting monomers can be repolymerized into new PET with identical properties to virgin plastic [13]. In spite of the benefits inherent to obtaining plastic with ideal properties, this process is inconvenient due to its high cost, and therefore, thermal treatments remain the most viable option. The discovery of the bacterium *Ideonella sakaiensis* 201-F6 and its ability to use PET as the main carbon source for growth [14] has garnered significant attention in recent years. *I. sakaiensis* employs two enzymes, *Is*PETase and *Is*MHETase, for breaking down PET into its building blocks (TPA and EG) (Figure 1). To date, *I. sakaiensis* represents a unique example where extracellular degradation of PET takes place in order to incorporate PET degradation products into cellular catabolism. *Is*PETase is a cutinase-like enzyme that catalyses the depolymerization of PET into mono(2-hydroxyethyl) terephthalic acid (MHET), with bis(2- hydroxyethyl) terephthalic acid (BHET) and TPA present in minor amounts as by-products [14] (Figure 1). A similar PET degrading activity has also been observed in cutinases from different organisms, including *Thermobifida fusca* (*Tfu*Cut) [15], *Thermobifida alba* (*Tal*Cut) [16], and *Thermofida cellulosilytica* (*Thc*Cut) [17], but these are not as efficient as *Is*PETase. The second enzyme, *Is*MHETase, catalyses the conversion of MHET into TPA and EG (Figure 1). The amounts of end-products generated in this pathway are strongly influenced by the reaction performed by *Is*PETase. For that reason, multiple efforts have focused on improving *Is*PETase activity and thermostability using rational approaches that integrate critical architectural elements found in enzymes of the cutinase and lipase families (Table 1). Nevertheless, the enzyme’s activity on highly crystalline PET, which is found in most soft drink bottles, has not reached the threshold for industrial applicability yet. Consequently, further investigation into novel modifications in the enzyme structure is still required.

In this study, a rational protein engineering based on an extensive analysis of the structure of *Is*PETase and different homologous enzymes was carried out with the aim to increase the enzyme activity. Molecular docking and molecular dynamic (MD) simulations suggested that three different single point mutations improve PET binding compared to the wild-type (WT) enzyme. Two of these mutations were introduced nearby the catalytic residue H237, which favour the binding affinity of PET through the introduction of further non-covalent interactions. A third mutation was introduced in the proximity of the active-site cleft in order to alter the flexibility of the loops that contour the active site. Examination of enzyme-treated PET films using scanning electron microscopy (SEM), atomic force microscopy (AFM), and differential scanning calorimetry (DSC) showed that the *Is*PETase mutant variants have an improved activity over crystalline PET films compared to the WT enzyme. This work could provide an important contribution to solving the environmental problem caused by plastic pollution through effective depolymerization of PET.

## 2. Materials and Methods

### 2.1. Molecular Docking

The PET substrate used for modelling purposes consisted of one subunit of BHET and one subunit of MHET (PET dimer). One initial three-dimensional conformer of the substrate was generated with Omega [24,25] and partial atomic charges of type am1-bcc were added to this conformer with Molcharge [26]. The structure of the *Is*PETase enzyme was obtained from the Protein Data Bank database (PDB code 6EQE). The PET dimer was docked into the active site of the enzyme with Gold [27]. The ChemPLP scoring function was selected for primary docking and scoring. A total of 30 docking solutions were generated and rescored with the GoldScore, ChemScore, and Astex Statistical Potential (ASP) scoring functions implemented in Gold. The best docking solution was selected from a consensus scoring scheme employed in previous research [28]. The binding mode of the products was generated by breaking the PET dimer and adopting the best docking solution into MHET and BHET.

### 2.2. Molecular Dynamic Simulations and Estimation of the Free Energies of Binding

Molecular dynamic (MD) simulations were performed with Amber 2022 [29] following a previously described protocol [30,31]. Briefly, the ff19SB and gaff2 force fields were used for the parametrization of the protein and ligands, respectively. Complexes were enclosed in truncated octahedron boxes and solvated with Optimal Point Charge (OPC) water molecules. Excess net charges were neutralized by adding sodium (Na^+^) and chloride (Cl^−^) counterions at a concentration of 150 mM [32]. Two stages of energy minimization were performed, with the solute restrained during the first of these and with no restrains during the second one. The energy minimized systems were gradually heated from 0 to 300 K, and equilibrated for 100 ps. The equilibrated systems were used as input to five short 4 ns MD simulations performed in the isobaric-isothermal (NPT) ensemble. Each MD replica was run with different random initial velocities for a better exploration of the complexes’ conformational space. The free energies of binding were estimated with the Molecular Mechanics-Generalized Born and Surface Area (MM-GBSA) method, as implemented in Amber 2022. For a complex, these calculations took place with 100 MD snapshots evenly drawn from all five MD replicas and covering the 1 ns–4 ns time interval. For each variant of the *Is*PETase enzyme, the free energies of binding were computed for the PET dimer and the reaction products.

### 2.3. Construction of the IsPETase Mutants

The *Is*PETase mutant variants were designed based on a comparison of the *Is*PETase substrate-binding site residues with those present in homologous enzymes, similar to that reported previously [18]. First, the amino acid sequences of different α/β hydrolases homologous to *Is*PETase were identified using PDBeFold and UniProt’s Basic Local Alignment Search Tool (BLAST) [33]. Then, the natural substitutions that occur in the residues that contour the substrate-binding site in the homologous enzymes were identified by multiple sequence alignment using ClustalOmega [34] and WebLogo [35] and assessed by MD simulations.

All of the constructs for the mutants were obtained using the QuickChange^®^ Site-Directed Mutagenesis kit (Agilent, Foster City, CA, USA) as described previously [36]. In brief, the wild-type *Is*PETase expression vector, which was previously codon optimized for expression in *Escherichia coli* and cloned into pET21b(+) with a C-terminus His-tag (pET21b(+)-*Is*PETase) [18], was obtained from Addgene. The pET21b(+)-*Is*PETase vector was amplified by polymerase chain reaction (PCR) using the *PfuUltra* polymerase with the primers I208V-F: 5′-GAGAATGATAGCTGGGCACCGGTGAAC-3′, I208V-R: 5′-GTTCACCGGTGCCCAGCTATCATTCTC-3′, N212A-F: 5′-ATTGCACCGGTGGCGAGCAGCGCGCTG-3′, N212A-R: 5′-CAGCGCGCTGCTCGCCACCGGTGCAAT-3′, S238Y-F: 5′-GGCGGTAGCCACTATTGTGCCAACTCT-3′, and S238Y-R: 5′-AGAGTTGGCACAATAGTGGCTACCGCC-3′. The parental plasmid was then digested with 1 unit of *Dpn I* restriction enzyme for 2 h at 37 °C, and the resultant mutant *Is*PETase-containing constructs were immediately transformed into *E. coli* DH5α chemically competent cells using the heat-shock method [37]. The introduction of each single point mutation was confirmed by Sanger sequencing.

### 2.4. Enzyme Expression and Purification

The wild type and mutant *Is*PETase variants were expressed and purified as reported previously [18,20,38] with some modifications. All expression vectors were transformed into *E. coli* BL21(DE3) Rosetta gami-B chemically competent cells. A single colony was transferred to 50 mL of Lysogeny Broth (LB) media supplemented with ampicillin (100 µg/mL) and incubated overnight at 37 °C with constant agitation (230 opm). This culture was transferred to 1 L of Terrific Broth media and incubated at 37 °C with constant agitation (200 opm) and, when the optical density (OD_600_) reached 2.0, the expression of the protein was induced with 0.1 mM isopropyl β-D-1-thiogalactopyranoside (IPTG) at 20 °C and 200 opm during 18–24 h. The bacterial cells were harvested by centrifugation at 4 °C, 3900× *g* for 20 min, and the cell pellet was collected and kept at −70 °C. All subsequent operations were performed at 4 °C. For lysis, the pellet was resuspended in buffer A (25 mM Tris, 150 mM sodium chloride, 20 mM imidazole, pH 7.5) supplemented with 0.5% Triton X-100 and 10 µL/mL 0.5 M ethylenediaminetetraacetic acid (EDTA) and DNase. The cell suspension was incubated for 15 min and then sonicated in an ultrasonic cell crusher (650 W, MRC, Harlow, UK for 10 × 10 s at a constant duty cycle, with rest intervals of 10 s. The cell debris was removed by centrifugation at 21,100× *g* for 1 h. The supernatant was loaded onto a HisTrap™HP column (Cytiva, Uppsala, Sweden), previously equilibrated with buffer A, using a FPLC system (ÄKTA Start, Cytiva, Uppsala, Sweden). The unbound proteins were removed with 10 column volumes (cv) of buffer A, and the enzyme was eluted with a gradient of 20–500 mM imidazole. The flow-through was collected and the fractions containing the enzyme were pooled and loaded onto a PD-10 desalting column (GE Health Care, Uppsala, Sweden) previously equilibrated with a buffer containing 25 mM Tris, 150 mM sodium chloride, and pH 7.5. The purified enzyme was stored in 50 µL aliquots at −70 °C. Enzyme purity was assessed by denaturing polyacrylamide gel electrophoresis (SDS-PAGE). All chemicals used were of analytical grade and were purchased from Sigma-Aldrich (Merck, Darmstadt, Germany), unless otherwise stated.

### 2.5. PET Degradation Assay

Circular PET films (6 mm diameter, 0.213 mm thick) cut from transparent commercial PET bottles were washed with sterile deionized water and dried at room temperature for 48 h before enzymatic treatment. The films were treated in 500 µL of a buffer containing 50 mM glycine-sodium hydroxide (NaOH), pH 9.4, with and without 500 nM enzyme. All samples were incubated at 30 °C for 72 h. The reaction was stopped by aqueous dilution with 20 mM phosphate, 12% dimethyl sulfoxide (DMSO) pH 2.5, and the precipitated enzyme was removed by centrifugation at 13,500× *g* for 10 min [14]. Then, the PET films were washed with 1% sodium dodecyl sulphate (SDS), distilled water, and ethanol. Finally, the films were air-dried for SEM, AFM, and DSC [18].

### 2.6. Scanning Electron Microscopy (SEM)

Dried samples were cleaned with nitrogen gas and then mounted on carbon tape and secured with silver tape on a SEM stub and then metalized using a sputter coater (Q150R ES, Quorum, Laughton, UK). Surface morphology of the control and *Is*PETase-treated samples was evaluated using a field emission scanning electron microscope (MIRA 3, TESCAN, Brno, Czech Republic) operating at 5 kV.

### 2.7. Atomic Force Microscopy (AFM)

Nanoscopic corrugation measurements were performed using an atomic force microscope (XE7, Park Systems, Santa Clara, CA, USA). Samples were attached to the sample holder with double-sided tape. Without any additional treatment, film surfaces were scanned in the non-contact mode under ambient conditions using PPP-CONTSCR commercial silicon cantilever tips (0.2 N/m, 25 kHz, <7 nm typical radius). All images were acquired at 512 × 512 pixels. AFM image edition was restricted to only a single polynomial levelling, performed using the Park System’s XEI software, version 5.1.6 build 1.

### 2.8. PET Crystallinity Assay

The thermal crystallinity of PET was assayed using a differential scanning calorimeter (DSC3 STAR^e^ System, Mettler Toledo, Urdorf, Switzerland). In summary, 6 mg of PET film was equilibrated at 25 °C, heated to 300 °C at 10 °C/min, held at 300 °C for 1 min, and cooled down to 25 °C at 10 °C/min. The experiment was carried out in a nitrogen (N_2_) atmosphere (50 mL/min). The percent crystallinity (%*X_c_*) of PET was determined as described previously [39] using Equation (1).

(1) $${\%X}_{c}=\;\left[\frac{{\Delta H}_{m}-{\Delta H}_{c}}{{\Delta H}_{m}^{o}}\right]*100$$

where ΔH*_m_* is the heat of melting, ΔH*_c_* is the heat of cold crystallization, and ΔH*_m_*° is the heat of melting of ideal PET crystals measured at the equilibrium melting point (140.1 J/g).

## 3. Results and Discussion

### 3.1. IsPETase Engineering for Enhancing PET-Degrading Activity

**Figure 2:**
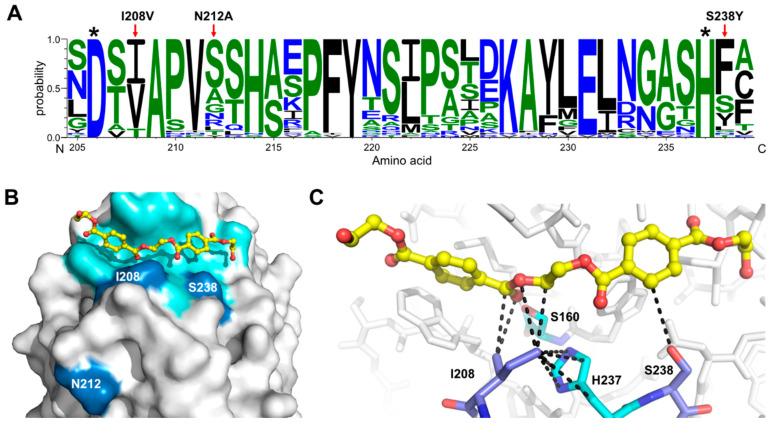
Single point mutations introduced in *Is*PETase. (**A**) Logo representation subalignment of *Is*PETase homologous enzymes. The * highlights the catalytic residues D206 and H237. (**B**) Connolly surface and PET modelled in *Is*PETase active site. The polypeptide, the residues forming contacts with the substrate and the mutations introduced in *Is*PETase are coloured light grey, cyan, and sky blue, respectively. (**C**) Snapshot of the MD simulation of the PET binding mode in the *Is*PETase active site. PET is represented in the ball and sticks model, while the protein amino acids are represented in the stick models. The carbon atoms are coloured yellow (PET), cyan (catalytic residues, S160 and H237), and sky blue (I208 and S238). Non-covalent interactions formed between the mutated residues, and the substrate or the polypeptide are represented by dashed lines.

To date, *Is*PETase constitutes the most efficient PET-degrading enzyme known. Compared to other enzymes that are able to hydrolyse PET, such as *Tfu*Cut, leaf-branch compost cutinase (LCC) and *Fusarium solani* cutinase (*Fs*Cut), this enzyme exhibits between 9 and 19.3-fold increased activity when using low-crystallinity (1.9%) PET films as the substrate [14]. However, *Is*PETase degrading activity is still insufficient for being used in the PET recycling industry, and biodegradation of PET pollution remains a major challenge due to the poor enzyme accessibility into the structure of highly crystallized PET used to manufacture beverage bottles. To overcome these problems, based on the previously reported crystal structure of *Is*PETase (PDB code 6EQE), molecular docking of PET and MD simulations, we designed three different single point mutations inspired in the architecture of 39 *Is*PETase homologous enzymes of the cutinase and lipase families (Figure 2A). MD simulations showed that PET binds to a cleft contoured by 14 residues located at the protein surface (Figure 2B). These include S160 and H237, which in consort with D206, conform the catalytic triad present in other enzymes of the α/β hydrolase superfamily [18] (Figure S1). We hypothesized that substitutions introduced nearby the catalytic residues could positively impact the binding affinity of the substrate, and consequently, the enzyme activity. S160 is flanked by W159 and W185, a highly conserved residue critical for enzyme activity (Table 1). On the other hand, H237 is flanked by I208 and S238, which also form part of the binding pocket (Figure 2B,C). A multiple sequence alignment (Figure S1) showed that most *Is*PETase homologous enzymes possess the W159H substitution, which confers an increased PET-degrading activity alone [19] or in combination with S238F [18], compared to the WT enzyme (Table 1). A greater variability was noted at positions I208 and S238, with two and six potential substitutions, respectively. Overall, we noted that the mutations I208V, S238F, and S238Y are more frequent in cutinases and lipases (Figure 2A). As stated above, the mutations W159H and S238F have previously been tested (Table 1); therefore, these were excluded in this study. A third position outside the binding pocket was also considered with the aim to alter the flexibility of the loop (E204-S214) that connects the *α5* helix and the *β7* strand, where D206 is located. The residue N212 was chosen for protein engineering due to its low level of conservation across all enzymes, and its position in the protein structure, with the side chain atoms exposed to the solvent (Figure 2A,B).

MD calculations served to discriminate the best mutant candidates regarding the values of free energy (ΔG) of substrate and products binding (Table 2), compared with those predicted when PET (−21.20 kcal/mol) or MHET (−17.94 kcal/mol) bind the WT. Interestingly, engineering *Is*PETase to adopt a cutinase/lipase-like active site through the introduction of I208V or S238Y mutations increased the binding affinity of PET by 20% (−25.50 kcal/mol) without altering the affinity of the reaction products. A similar result was observed when the asparagine at position 212 was mutated to alanine (ΔG PET binding = −28.36 kcal/mol) (Table 2). Thus, we generated the *Is*PETase I208V, S238Y, and N212A variants using mutagenic PCR. The enzymes were then expressed in *E. coli* and isolated by affinity chromatography.

### 3.2. Modified IsPETase Exhibits Increased PET-Degrading Activity over Highly Crystallized PET

As mentioned above, in the original research report where *Is*PETase was isolated and characterized, Yoshida et al. [14] used low crystallinity (1.9%) PET to examine the ability of the enzyme to digest this polymer. In contrast, PET waste remains a challenge for recycling because it usually shows a much higher degree of crystallinity, which reduces the enzyme’s esterase activity. Here, we performed enzyme-mediated PET degradation assays using coupons (6 mm diameter, 0.213 mm thick) mechanically cut from commercial soft-drink PET bottles with 30.88% crystallinity (determined by DSC).

**Figure 3:**
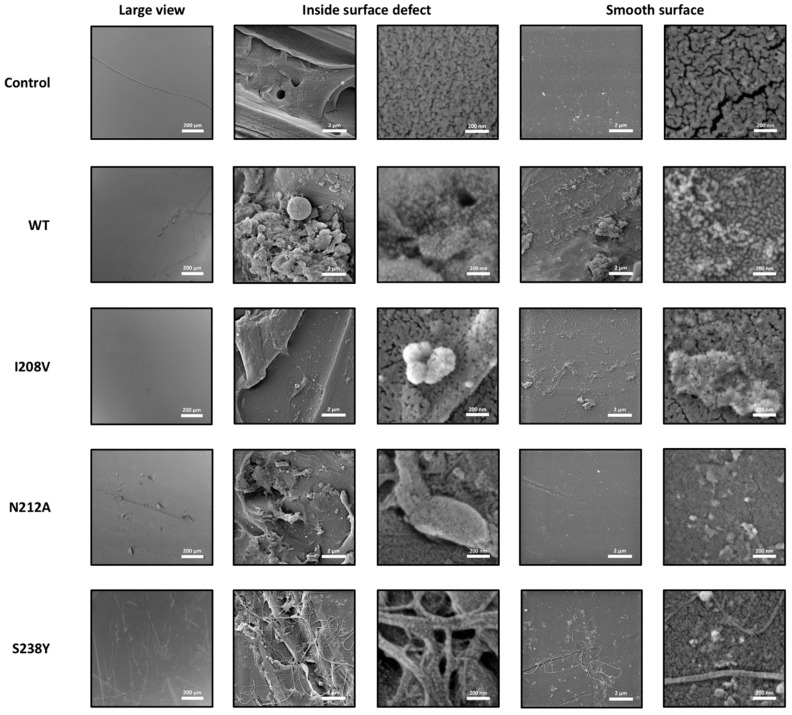
SEM micrographs of PET films degraded by *Is*PETase WT and mutant variants. All films were incubated in buffer 50 mM glycine-NaOH pH 9.4, with or without enzyme, at 30 °C for 72 h.

SEM microphotographs of the control sample and those treated with the WT and mutant enzymes were acquired at different magnifications to evaluate their surface morphology. SEM microphotographs showed the existence of surface defects in the form of channels in all the samples (Figure 3, top left corner), possibly originated from the manipulation of the films. Considering this fact, the surface of the samples was evaluated inside and outside the surface defects. It could be noticed that the control sample exhibits a smoother surface (first row in Figure 3) compared to the enzyme-treated samples (second to fifth rows in Figure 3). The enzymes induced a granular texture and pitting on the PET film surface, a clear sign that these were degraded the polymer. These findings indicated that the enzyme could break down PET that is highly crystalline, and that the process is comparable to when the enzyme breaks down PET with lower crystallinity levels (1.9% [14] or 14.8% crystallinity [18]). In addition, we observed that, in all enzyme-treated films, there is a greater change in the morphology inside the surface defects. Therefore, we inferred that the enzyme activity is initiated and accentuated in those parts that previously presented surface defects, most likely due to favoured polyester chain flexibility [40] induced by mechanical stress. Amongst the three mutants, PET films treated with the I208V variant showed fewer changes in the morphology of the PET surface compared to the WT and the N212 and S238Y variants, suggesting a decreased PET-degrading activity. Interestingly, the S238Y mutant induced a severe degradation of the PET film surface and the formation of microscopic PET filaments in the order of several hundred of nanometers wide (typically around 200 nm) inside and outside the surface defects (bottom row in Figure 3). This finding constitutes the first report where an *Is*PETase mutant variant produced this type of degradation of PET, since all previous data found in the literature has only shown a prominent pitting [14,18].

**Figure 4:**
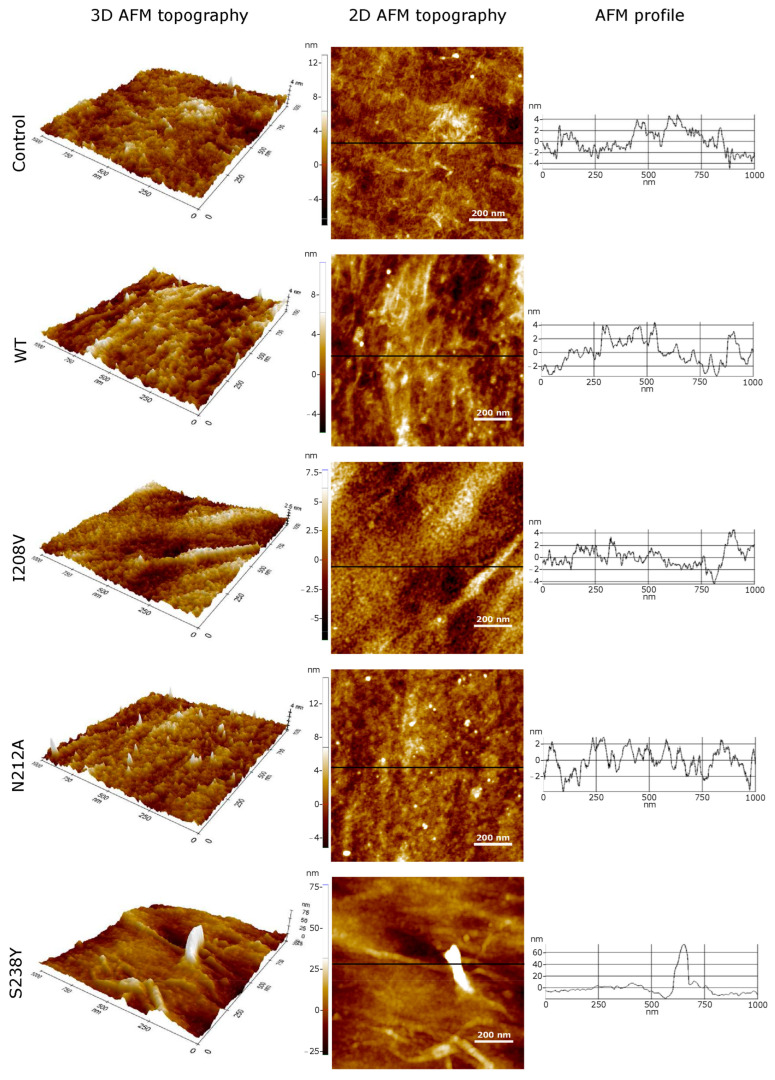
AFM surface topographic 3D and 2D characterizations of PET films degraded by *Is*PETase WT and mutant variants. The profiles extracted from the depicted solid lines reveal the height differences.

A similar type of PET surface morphology modifications was observed in AFM characterizations. AFM confirmed SEM surface observations by permitting topographic profile measurements on samples without any coating. We analyzed the PET film surface using 2D and 3D views and the AFM profile. The 2D AFM topographic images represent zenithal views of the height data recorded at each sample using a colour code to describe the elevation values. The 3D AFM images display the same surface using a three-dimensional perspective. Profile diagrams depict linear altitudes measured along the line highlighted on the 2D corresponding picture. A comparison at the nanoscale between enzyme-treated samples and the buffer-treated PET films revealed important topographic modifications of the PET surfaces in agreement with a previous report [41]. These can be observed at the profile corrugation contrast between control and sample scans presented in Figure 4. Furthermore, the results show that the WT and N212A mutant apparently attack the PET sample heterogeneously, degrading some regions more intensively than others and producing a hollowed surface (see WT and N212A images in Figure 4). This suggests that, at the nanoscale, PET bottles may show some degree of heterogeneity, and *Is*PETase could attack first spots with more flexible polymer chains exposed on the original PET surface. On the contrary, the I208V mutant may damage the PET more homogeneously, creating a flatter but granular structure, as displayed in picture I208I of Figure 4. In the particular case of S238Y, filamentous degradation protrusions beyond 17 times above the highest corrugation measured on the other samples were detected, in agreement with SEM images shown previously. In addition, PET surfaces treated with the S238Y variant exhibit hollows 15-fold deeper compared to those observed for the WT-treated PET film. In a previous report [41], AFM observations suggested that the enzymatic degradation of PET induces the corrugation of the film surface in a time-dependent manner and attributed this change to the accumulation of the BHET product on the polymer surface. Here, our SEM and AFM measurements suggest a similar type of corrugation for all enzyme variants, except for the S238Y mutant that induced a hollowed surface. This could indicate that the activity of the S238Y mutant does not produce significant amounts of BHET and principally yields MHET or TPA.

**Figure 5:**
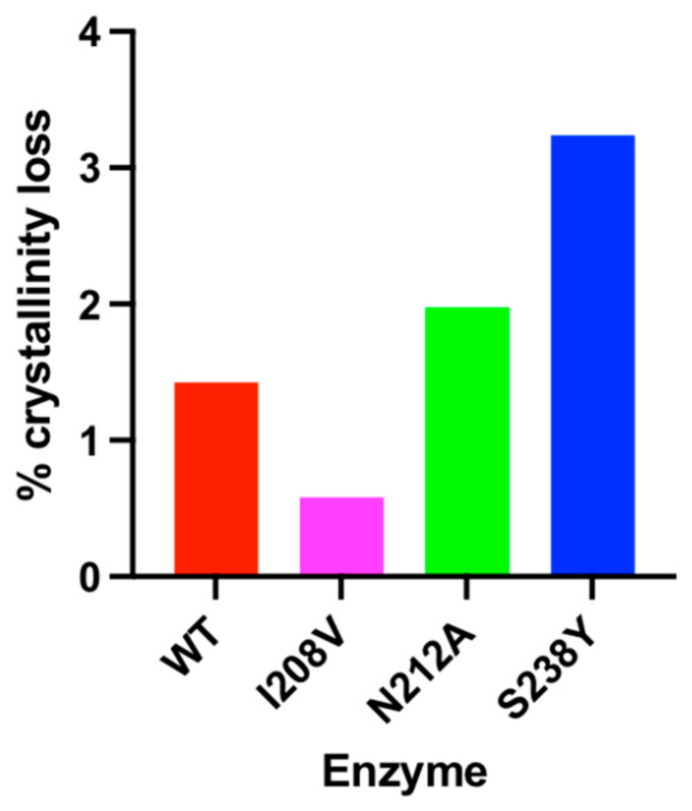
Engineered *Is*PETase provoke changes in the crystalline structure of PET. The crystallinity of enzyme-treated PET was analysed by DSC.

In order to further characterize the PET-degrading activity of the mutant variants, changes on the structure of the polymer were analysed using DSC. Similar to that reported previously [18], *Is*PETase induced a reduction in the crystallinity of PET. All three mutant variants performed differently than the WT enzyme under this observation, with the S238Y and the N212A mutants showing 3.3- and 1.4-fold increased activity, respectively. In contrast, the I208V mutant barely altered the crystalline structure of PET (Figure 5).

**Figure 6:**
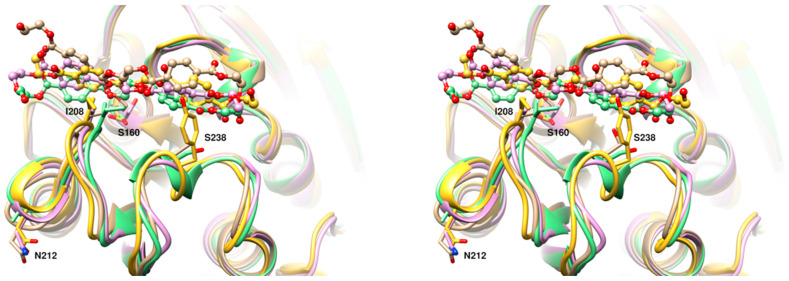
Stereoview of the superposition of *Is*PETase WT (tan) and the mutant variants, I208V (magenta), N212A (green), and S238Y (yellow) with PET bound. PET is represented as balls and sticks, while the mutated residues are depicted as sticks models. The catalytic residue S160 is also shown.

These results provided some clues about the mechanism of action of the *Is*PETase mutant variants created in this study. First, our MD calculations predicted an increased binding affinity of PET to the I208V mutant compared to the WT enzyme (Table 2). However, in vitro assays showed a decreased degrading activity. A possible explanation to these results is that the substitution of isoleucine for valine disrupts several non-covalent interactions formed between the Cδ of the isoleucine and the side chain atoms of H237, a key residue part of the catalytic triad (Figure 2 and Figure 6). These interactions orient the imidazole ring of H237 towards S160, allowing its deprotonation [18,20]. Thus, the substitution I208V may have contributed to a greater flexibility of H237, reducing the enzyme turnover. On the other hand, the S238Y mutation allows the aromatic side chain of the introduced tyrosine residue to position favourably for a π-π stacking interaction with the PET substrate (Figure 6), similar to that observed when this residue is mutated to phenylalanine [18,42]. This interaction could lead to a better affinity of the mutated enzyme for PET relative to the WT serine residue. Finally, despite N212 not being directly involved in the recognition of the substrate, its mutation to alanine could have a favourable structural impact on the enzyme’s mechanism. In the WT enzyme, N212 is located at the beginning of a α-helix and points to the solvent. The N212A mutation could make this portion of the helix move closer to the rest of the protein, affecting the loop 204–210 that we speculate moves to narrow the PET binding channel. In addition, the displacement of the 204–210 loop could position the catalytic residue D206 in a more favourable location for catalysis (Figure 6).

## 4. Conclusions

In the present study, *Is*PETase mutants were engineered for enhancing PET-degrading activity using a computational rational design approach of the enzyme’s active site. This was achieved by comparing the 3D structure of *Is*PETase with that of homologous esterase-like enzymes (cutinases and lipases). In particular, the S238Y substitution provoked a substantially increased PET-degrading activity, expressed in the form of modifications of the PET surface and crystallinity loss, when using highly crystalline PET from commercial PET bottles as substrate. To date, PET recycling is mostly conducted via thermal treatments that produce dangerous gas emissions, linked to severe health and environmental issues. The results presented here prove the concept that taking advantage of *Is*PETase’s recent evolution to adopt a PET-degrading activity could facilitate PET pollution management in a sustainable manner and that the introduction of single-point mutations in the enzyme architecture can significantly impact its applicability in the recycling industry. Moreover, the methodology presented in this study provides a first step towards rapidly identifying new potential mutations of the enzyme for enhancing its activity. This investigation adds new information about the important role of I208 in catalysis and contributes to understanding the mechanism of PET degradation by *Is*PETase.

It has been shown that treatment of the PET surface with physical or chemical agents (detergents, organic solvents, heat, or alkali) enhances *Is*PETase activity, most likely by promoting an amorphous conformation of the polymer chains and improving the enzyme’s accessibility to the substrate. Here, we identified that the performance of *Is*PETase is improved when PET suffers mechanical stress over its smooth surface. This finding could be highly important in the recycling context since it provides new information for designing pre-treatments for the enzymatic treatment of PET pollution that accumulates in landfills and the environment.

## Figures and Tables

**Table 1:** Mutations of *Is*PETase structure for modifying its PET-degrading activity.

Mutation	Effect on Enzymatic Activity		Method	Substrate	Ref.
S238F/W159H	4.13% higher than wild type		Absolute crystallinity loss	PET 14.8 ± 0.2% crystallinity	[18]
W185A	highly impaired performance relative to wild type				
S160A	Not detected	Disrupt the catalysis process	Relative activity towards MHET and TPA production	PET drinking bottle	[19]
D206A					
H237A					
W159A	Increased	Influence the substrate binding			
W159H	Increased				
M161A	Decreased				
W185A	Decreased				
A209I	No change observed				
Q119A	Decreased				
S214H	Increased				
S238F	Decreased				
W97L	Decreased	Change the hydrophobic property			
Q182L	No change				
R123A	Decreased				
N241A	Decreased				
S160A	Decreased		Expressed by production levels of MHET and TPA.	BHET	[20]
R132G	Decreased				
C203S	Decreased				
C239S	Decreased				
W185A	Decreased				
S214H	Decreased				
I208A	Decreased				
W159A	Decreased				
W159H	Decreased				
M161A	Decreased				
Y87A	80.73% MHET production; TPA production decreased				
T88A	Full activity in producing MHET; TPA production decreased				
R61A	Increased 1.6 times wild type activity		Expressed by kinetic parameters (k_cat_/K_M_)	PET film	[21]
L88F	Increased 2.0 times wild type activity				
I179F	Increased 15.0 times wild type activity				
S178T	Decreased to 29.7% of the activity of wild type				
S209V	Decreased to 38.2% of the activity of wild type				
S160A	Almost complete loss		Expressed hydrolytic activity	BHET	[22]
D206A	Almost complete loss				
H237A	Almost complete loss				
Y87A	5% hydrolytic activity				
M161A	52% hydrolytic activity				
W185A	5% hydrolytic activity				
I208A	46% hydrolytic activity				
W159A	8% hydrolytic activity				
S238A	Similar hydrolytic activity				
N241A	18% hydrolytic activity				
R280A	Similar hydrolytic activity, increased thermostability and PET degradation activity by 14-fold at 40 degrees Celsius; when associated with E-121 and H-186.				
W159H	Dramatically decreased				
S238F	Dramatically decreased				
C203A/C239A	Dramatically decreased				
S93M	Increases activity towards 1-naphthyl butyrate		Expressed hydrolytic activity	1-naphthyl butyrate	[23]
W159F					
N241F					

**Table 2:** Binding free energy calculations of substrate and products in complex with *Is*PETase.

*Is*PETase	ΔG (kcal/mol)	
	PET	Products
WT	−21.20	−17.94
I208V	−25.50	−18.58
N212A	−28.36	−14.88
S238Y	−25.50	−17.84

## Data Availability

Not applicable.

## Supplementary Materials

The following supporting information can be downloaded at: https://www.mdpi.com/article/10.3390/polym15071779/s1, Figure S1: Multiple sequence alignment of IsPETase homologous enzymes.
