# Supplementary material for: Degradation of PET Bottles by an Engineered Ideonella sakaiensis PETase
Source: Polymers (Basel). 2023 Apr 3;15(7):1779. doi: 10.3390/polym15071779 (PMC10098701; doi:10.3390/polym15071779)
Supplement: Supplementary file 1 [file polymers-15-01779-s001.zip › polymers-2285185-supplementary.pdf]

Supplementary materials

```
IsPETase             -------------------------------MNFPR-----ASRLMQAAVLGGLMAVSAA     24
Sblipase             ---------------------------MPPDC--VL-----PRRLAAAALLASATLVPLS     26
RgDLH                --------------------------MFGKLPFAR------------ASLAVGALLLSAA     22
RdPETase             -----------------------------MNFTTL-----WRRHARACA--LAASVV---     21
AqPET_hydrolase      -----------------------------MKTT-V-----S--RIKALS--LAAAL----     17
MsPET_hydrolase      -----------------------------MRNSTTTRPRSSISTLARLA--VTAVLAGGA     29
MesCutinase          -----------------------------MPSPTTIRPRSLVRAVAGLT--TAVLLATGG     29
MgCutinase           -----------------------------MRSPTTTRTHTLRSAVARFA--VAAVLAAGG     29
Mcα_βHydrolase       ---------------------------------------------------MAAVLAAGG     9
MmLipaseT            -----------------------------MRSTTTTRPRSL-TLLARLA--MTAVLAAGG     28
MaCutinase           -----------------------------MPVSTTTRPRSVLSRIGRTA--MAAVLVAGG     29
Meα_βHydrolase       -----------------------------MSSATTTRTRPLAGRVARLA--LAATLSATA     29
MaLipaseT            -------------------------------MPTTIRSRTSVRALARLA--VAALLATGG     27
McCutinase           -----------------------------MRSPTTTRPRSAVRTVAGLA--MAAVLAAGG     29
MefCutinase          -----------------------------MSSPTTTVRPRPAVRLAGLA--VAAVLATAG     29
DmLipaseT            ----------MSNGYS---ACPAPYDHRGMRKHASLLA-ALPVLLAACG--TTTSTLTTP     44
CcLipase             MPLVATLGLALAAGSLAPSASAAAGDPATVETPPVV---SQRAIDEHAA---TEPQLSLD     54
PmDLH                ---------------------------MVLRNPRSP---RWRRALGVV----VATLALTG     26
AaPET_hydrolase      ----------------------------------------MRKRLTGAA---LA-IALGA     16
SsLipase             ------------------------------------------------------------     0
Ssα_βHydrolase       -----------------------------MR---------LRRLIPSLI---PV--LALS     17
Asα_βHydrolase       -----------------------------MRN-P------VKHLLTAAT---LVATLVAA     21
AmLipaseT            -----------------------------MR---------LRALFPAMA---L--LAASA     17
AoCutinase           -----------------------------MR---------IRSIFCALS---T--SALLL     17
Ksα_βHydrolase       ----------------------------MLEGRYMK-----TVRFNTAAAVFTSALLSSQ     27
Kaα_βHydrolase       ----------------------------------MK-----LDILKPLIVTLVAGMLSSA     21
AxCutinase           -----------------------------MKTTTLI-----KSTLC-----IISLSFSLS     21
OaLipase             ----------------------------------MN-----KSILKKLSFGTSVLLVSMN     21
AeDLH                ------------------------------MSSIIK-----RKLASLFAF-SAAMLLSTS     24
HsCutinase           -----------------------------MKTQSIG-----KTLFSVVA--ASALLFSTS     24
MdCutinase           ------------------------------------------------------------     0
PyLipaseT            ------------------------------MKHNMH-----KLALSLLA--TGALMFSSA     23
TaCutinase_est2      ------------------------------------------------------MRGSHH     6
TcCutinase_2         ------------------------------------------------------------     0
TcCutinase_1         ------------------------------------------------------------     0
TfCutinase           ------------------------------------------------------------     0
Stsα_βHydrolase      ------------------------------------------------------------     0
PsLipase             ------------------------------------------------------------     0
MmaLipaseT           ------------------------------------------------------------     0
HpDLH                -------------------------------MPFNK-----KSVLALCG--AGALLFSMS     22


IsPETase             AT-----------------------AQTNPYARGPNPTAASLEA-SAGPFTVRSFTVSRP     60
Sblipase             AA-----------------------AQTNPYQRGPDPTTRDLED-SRGPFRYASTNVRSP     62
RgDLH                AV-----------------------AQTNPYQRGPDPTVSSLEA-TRGPFSTSSFTVSRP     58
RdPETase             -L-------------------AMSATSGWAQQTGPDPTSASLNA-TAGPFAVSTATVT--     58
AqPET_hydrolase      ---------------------AAATLATPALSQTSAPTSASLNA-SSGPLSVSTASVT--     53
MsPET_hydrolase      ALAV---------------PAA-AQAADNPYERGPAPTSATLEA-SRGPFATSSFSVSSL     72
MesCutinase          VLAA---------------ASG-AQAADNPYERGPNPTTAILEA-SRGPFATASQTVSSL     72
MgCutinase           VLAP---------------SPA-AQAAANPYERGPAPTSAMLEA-SRGPFATSSTRVSSL     72
Mcα_βHydrolase       VVAT---------------SSA-AQ-AANPYERGPAPTHAILEA-SRGPFATSSLNVSSL     51
MmLipaseT            VVAT---------------SSA-AQ-AANPYERGPAPTNAILEA-SRGPFATSSTTVSSL     70
MaCutinase           ALAA---------------SSGPAQAADNPYERGPAPTVALLEA-SRGPFTTASQNVSSL     73
Meα_βHydrolase       AVAA---------------P-A-AAQAASPYERGPNPTVALLEA-SRGPFATSSQSVSSW     71
MaLipaseT            ALAV---------------PVA--ANAASPYERGPAPTNAILEA-SRGPFATSSINVSSL     69
McCutinase           VLAT---------------SPG-AQAAENPYERGPAPTSAILEA-SRGPFATSSQSVSSW     72
MefCutinase          AFAA---------------PPA-AQAAANPYERGPAPTVALLEA-SRGPFATASQSVSSL     72
DmLipaseT            AVTP---------------Q-TLSGAATNPYERGPAPTTALLEA-ARGPYATASTTVPRS     87
CcLipase             APAA---------------L-PPVTIAASPYERGPAPTVASIEA-ARGPFAVAQTTVSRF     97
PmDLH                GLTG---------------T-QVAAAQDNPYQRGPDPTEQSIEA-PRGPFSVAETRVS-S     68
AaPET_hydrolase      ATFA---------------G-PAASAAENPYERGPDPTTSSIEA-PTGPFSTAETTVS-S     58
SsLipase             --LT---------------G-PGARAADNPYERGPAPTTASIEA-SRGPYSVSETSVSSL     41
Ssα_βHydrolase       -LAG---------------V-TPATAAEAEYRRGPDPTTAGIEA-PRGPFTAETTTVSRS     59
Asα_βHydrolase       LVAV---------------P-AGPALAANPYERGPAPTTASIEA-SLGSFAIAQVTVARS     64
AmLipaseT            -VAA---------------P-PAALAAASPYERGPAPTTASVEA-ARGPFATATATV--S     57
AoCutinase           VGAT---------------A-PAAQAAANPYERGPAPTISSIQA-SRGPFATASVTVARS     60
Ksα_βHydrolase       VFAITDDPVDPVDPVDPPS---------SGTVRGPDPTLSALESTRSGPYSVRTENVSNL     78
Kaα_βHydrolase       AFAITPSPTPTPDPTPNPSPDPGSCSGAECYIRGPNPTVRALEA-DDGPYSVRTTNVSSF     80
AxCutinase           AFGGGSGGGSTG----DDLPLPDTCTSNCGYELGPNPSLSFVSA-AQGPQPVSTVSVSSS     76
OaLipase             ALSWTPSPTPNP----DPIPDPTPCQDDCDFTRGPNPTPSSLEA-STGPYSVATRSVASS     76
AeDLH                VWSMNPG------------DVPDTCEGDCGYARGPDPTESFLEA-DSGPYTIATSNVSSL     71
HsCutinase           VMASNPTPPPP-----PPPSEPTDPGAGSPYQRGPNPTTSFLEG-SRGQYRVATSNVSSF     78
MdCutinase           ------------------------------------------ES-SSGPFSVRTSNVSSF     17
PyLipaseT            ALASNPA-----------PDTGSGDGSSSQYQIGPDPTVSFLEA-SSGPFSVRTSNVSSL     71
TaCutinase_est2      -HHH---------------HGSAPAQAANPYERGPNPTESMLEA-RSGPFSVSEERASRF     49
```

```
TcCutinase_2          --------------------------MANPYERGPNPTDALLEA-RSGPFSVSEERASRF     33
TcCutinase_1          --------------------------MANPYERGPNPTDALLEA-SSGPFSVSEENVSRL     33
TfCutinase            ---------------------------ANPYERGPNPTDALLEA-SSGPFSVSEENVSRL     32
Stsα_βHydrolase       --------------------------AANPYERGPAPTNASIEA-SRGPYATSQTSVSSL     33
PsLipase              ------------APLPDTP-------------GAPFPAVANF--DRSGPYTVSSQSEGP-     32
MmaLipaseT            --------------------------MANPYERGPDPTESSIEA-VRGPFAVAQTTVSRL     33
HpDLH                 ALANNPA-----------PTDPGDSGGGSAYQRGPDPSVSFLEA-DRGQYSVRSSRVSSL     70
                                                                     *

IsPETase              --SGYGAGTVYYPTNA-GGTVGAIAIV--PGYTARQSSIKWWGPRLASHGFVVITIDTNS     115
Sblipase              --SGYGAGTIYYPTDV-SGSVGAVAVV--PGYLARQSSIRWWGPRLASHGFVVITLDTRS     117
RgDLH                 --SGYGAGTVYYPTNA-GGKVGAIAVV--PGYTARQSSINWWGPRLASHGFVVITIDTNS     113
RdPETase              SPVGFGGGTIYYPTI--AGQYGVVALS--PGFTATQSSVAWLGRRIATHGFVVVTINTNS     114
AqPET_hydrolase       LPRGFGGGTIYYPTV--AAKYGVIAIS--PGFTATQSSIAWLGRRIATHGFVVITINTNT     109
MsPET_hydrolase       LVSGFGGGVVYYPTSTSEGTFGAVAIS--PGFTASWSSLDWLGPRIASHGFVVIGIETNT     130
MesCutinase           SVSGFGGGVIYYPTSTSEGTFGAIAIS--PGFTAYWSSISWLGPRLASHGFVVIGIETNS     130
MgCutinase            SVSGFGGGVIYYPTSTSEGTFGGIAIS--PGYTASWSSIDWLGPRLASHGFVVIGIETNS     130
Mcα_βHydrolase        -VSGFGGGVIYYPTSTSQGTFGAVAIS--PGYTAAWSSIDWLGPRIASHGFVVIGIETNS     108
MmLipaseT             -VSGFGGGVIYYPTSTSEGTFGAVAIS--PGYTASWSSLDWLGPRIASHGFVVIGIDTIT     127
MaCutinase            SVTGFGGGVIYYPTDTSQGTFGAIAIS--PGYTAAWSSISWLGPRIASHGFVVIGIETNS     131
Meα_βHydrolase        SVTGFGGGVIYYPTTTSEGTFGAVAIS--PGYTASWSSISWLGPRLASHGFVVIGIETNS     129
MaLipaseT             SVTGFGGGVIYYPTSTAEGTFGAVAIS--PGYTASWSSLSWLGPRIASHGFVVIGIETNT     127
McCutinase            SVTGFGGGVIYYPTSTSEGTFGAIAIS--PGYTAAWSSISWLGPRIASHGFVVIGIETNS     130
MefCutinase           SVTGFGGGVIYYPTSTAEGTFGAVAIS--PGYTAAWSSIDWLGPRIASHGFVVIGIETNT     130
DmLipaseT             SVSDFGGATIYYPTSTADGTFGGVAIS--PGYTGTQASVAWLGPRLASHGFVVIVIDTLS     145
CcLipase              AASGFGGGTITYPTSTAEGTFGAVAIS--PGYTAAQSSISWLGPRLASQGFVVITIDTNS     155
PmDLH                 LVSGFGGGTIYYPTDTSQGTFGAVAIA--PGYTADQSSMSWYGPRLASQGFVVFTIDTNS     126
AaPET_hydrolase       LVSGFGGGTVYYPTSTAEGTYGALAIS--PGFTATQSSISWMGRRLASQGFVVITIDTNT     116
SsLipase              AVSGFGGGTIYYPTSTADGTFGAVAVS--PGYTGTQSSIAWLGPRLASQGFVVFTIDTLT     99
Ssα_βHydrolase        SASGFGGGTIYYPTSTAEGTFGAVAIS--PGFTGTQSSISWYGPRLASHGFVVITIDTNS     117
Asα_βHydrolase        SVSGFGGGTIYYPTSTAEGTFGAVAAS--PGFTASQSSVAWYGPRLASQGFVVITIDTLS     122
AmLipaseT             SVSGFGGGTVYYPTSTAEGTFGAVAIS--PGFTATQSSVAWLGPRLASQGFVVITINTLS     115
AoCutinase            SVSGFGGGTIYYPTSTAEGTFGAVAIS--PGFTASQSSVSWLGPRVASQGFVVITIDTLS     118
Ksα_βHydrolase        SASGFGGGTIHYPTNA-GENMGAIAVI--PGYVSYESSIEWWGPRLASWGFVVITIDTNT     135
Kaα_βHydrolase        -VSGFGGGTIHYPVGT-EGKMGAIAVI--PGYVSYESSIRWWGSRLASWGFVVITIDTNT     136
AxCutinase            -VDGFGGGTIYYPSNI-SQEMAAISIA--PGFTNTQSAIAWWGPLLASHGFVVITIDTNS     132
OaLipase              -VSGFGGGTLHYPTNT-TGTMGAIAVV--PGFLLQESSIDFWGPKLASHGFVVITISANS     132
AeDLH                 -VRGFGGGTIYYPVNA-EGTMAAIVVI--PGYMSYQSSISFWGPRLASHGFVVMTIDTNR     127
HsCutinase            -VSGFGGGTIHYPSNA-SGSMAAIVVI--PGYVSSESSIQWWGPKLASYGFVVMTIDTNS     134
MdCutinase            -VSGFGGGTIHYPTGT-TGTMAAIVVI--PGFASAESSIAWWGPKLASHGFVVMTIDTNS     73
PyLipaseT             -VSGFGGGTIHYPTGV-NRPMAAIVVI--PGFVSAESSIDWWGPKLASYGFVVMTIGTNS     127
TaCutinase_est2       GADGFGGGTIYYPRE--NNTYGAIAIS--PGYTGTQSSIAWLGERIASHGFVVIAIDTNT     105
TcCutinase_2          GADGFGGGTIYYPRE--NNTYGAVAIS--PGYTGTQASVAWLGERIASHGFVVITIDTNT     89
TcCutinase_1          SASGFGGGTIYYPRE--NNTYGAVAIS--PGYTGTEASIAWLGERIASHGFVVITIDTIT     89
TfCutinase            SASGFGGGTIYYPRE--NNTYGAVAIS--PGYTGTEASIAWLGERIASHGFVVITIDTIT     88
Stsα_βHydrolase       VASGFGGGTIYYPTSTADGTFGAVVIS--PGFTAYQSSIAWLGPRLASQGFVVFTIDTNT     91
PsLipase              ------SCRIYRPRDLGQGGVRHPVILWGNGTGAGPSTYAGLLSHWASHGFVVAAAETSN     86
MmaLipaseT            QADGFGGGTIYYPTDTSQGTFGAVAIS--PGFTAGQESIAWLGPRIASQGFVVITIDTIT     91
HpDLH                 -VSGFGGGTIYYPTGT-TGTMGAVVVI--PGFVSAESSIDWWGPKLASYGFVVMTIDTNT     126
                            .  :  *                 *      :        *: ****   :

                                                          W159 S160
                                                             ↓ ↓
IsPETase              TLDQPSSRSSQQMAALRQVASLNGTSSSPIYGKVDTARMGVMGWSMGGGGSLISAANNPS     175
Sblipase              TSDQPASRSAQQMAALRQVVALSETRSSPIYGKVDPNRLAVMGWSMGGGGTLISARDNPS     177
RgDLH                 TLDQPSSRSSQQMAALRQVVSLAGTSSSPIYNKVDTARLGVMGWSMGGGGSLISAKNNPS     173
RdPETase              TLDQPASRANQLIAALNYVANS---ASTTVRSRVDASRRAVGGHSMGGGGSLIAAQNNPS     171
AqPET_hydrolase       TLDQPDSRATQLVAALNHVLNS---SGTAVRSRIDPARLAVAGHSMGGGGSLIAARDNPT     166
MsPET_hydrolase       RLDQPASRGRQLLAALDWLTQR-----SSVRGRIDATRLAVAGHSMGGGGSLEAAVSRPS     185
MesCutinase           TLDQPDSRGRQLLAALDYLVQR-----SSVRTRIDGTRLAVAGHSMGGGGSLEASVSRPS     185
MgCutinase            RFDQPASRGRQLLAALDYLVER-----SSVRSRVDGSRLAVSGHSMGGGGSLEAAAERPS     185
Mcα_βHydrolase        RLDQPASRGRQLLAALDYLVER-----SSVRSRVDGSRLAVAGHSMGGGGSLEAAASRPS     163
MmLipaseT             RLDQPSSRGRQLLAALDYLTER-----SSVRSRIDSSRLAVAGHSMGGGGSLEAAASRPS     182
MaCutinase            RLDQPDSRGRQLLAALDYLVER-----SSVRTRIDGTRLAVAGHSMGGGGSLEAAASRPS     186
Meα_βHydrolase        RFDQPDSRGRQLLAALDYLTER-----SSVRGRIDASRQAVAGHSMGGGGSLEAASSRPS     184
MaLipaseT             RLDQPDSRGRQLLAALDYLTQR-----SSVRGRIDSSRLAVAGHSMGGGGSLEAAAARPS     182
McCutinase            RLDQPDSRGRQLLAALDYLVER-----SSVRGRIDGTRLAVAGHSMGGGGSLEAAVARPS     185
MefCutinase           RLDQPDSRGRQLLAALDYLTQR-----SSVRGRIDASRLAVSGHSMGGGGSLEAAVARPS     185
DmLipaseT             RYDYPSSRGDQLRAALRYLTTS-----SAVRTRVDATRLAVMGHSMGGGGALEAAKDNPA     200
CcLipase              RYDQPSSRGDQLRAALAYLTGS-----SSVRGRIDASRLAVMGHSMGGGGALEAAKDNPA     210
PmDLH                 RYDQPASRGDQLLAALDYLVQR-----SSVRDRIDGQRLGVMGHSMGGGGSLEAADDRPG     181
AaPET_hydrolase       IYDQPASRGDQLLAALDYLTQQ---SPSTVRSKIDPSRLAVAGHSMGGGGSLEASVKRPS     173
SsLipase              TLDQPDSRGRQLLAALDYLTTR-----SSVRGRVDSTRLGVMGHSMGGGGSLEAAKSRPS     154
Ssα_βHydrolase        TLDAPDSRATQLLAALDYLTQR-----STVRTRIDASRLGVMGHSMGGGGSLRAAEQRPA     172
Asα_βHydrolase        TLDSPASRGTQLLAALDYLTTS-----SAVRTRIDATRLGVMGHSMGGGGSLAASRTRPA     177
AmLipaseT             TLDQPDSRGAQLLAALDYLTTS-----SSVRTRIDASRLGVMGHSMGGGGSLRATVSRPS     170
AoCutinase            TLDQPDSRGTQLLAALDYVTNS-----SSVRTRVDATRLGVMGHSMGGGGSLSASRTRPT     173
Ksα_βHydrolase        IYDQPDSRADQLSAALDHLIDESGSSSSPISGLVDASRLGVIGWSMGGGGTLKLATE-RN     194
Kaα_βHydrolase        IYDQPDSRANQLSAALDYVIAQSNSRNSSISGMVDSNRLGVIGWSMGGGGSLKLSTQ-RT     195
```

```
AxCutinase            RFDQPDSRSRQLDSALSYLISQSDSSSSSISGLVDENRLATMGFSMGGGGSLQSASR-NR      191
OaLipase              GFDQPASRATQLGRALDYVINQSNGSNSPISGMVDTTRLGVVGWSMGGGGALQLASG-DR      191
AeDLH                 ISDQPPSRRDQIEAALEYLVDQSNSSRSPINGMVDPNRLGAVGWSMGGGGTLRLAAD-DG      186
HsCutinase            GFDQPPSRARQINSALDYLVDQNNSRNSPVRGMIDTQRLGVIGWSMGGGGTIRVAGE-GR      193
MdCutinase            GFDQPGSRATQINNALDYLIDQNSSSSSPINGMVDTSRLGAVGWSMGGGGTLRVAAE-GR      132
PyLipaseT             GFDQPPSRARQINNALDYLVAQNTNSRSPVNGMIDTNRLGAMGWSMGGGGTLRVAEE-GR      186
TaCutinase_est2       TLDQPDSRARQLNAALDYMLTD---ASSAVRNRIDASRLAVMGHSMGGGGTLRLASQRPD      162
TcCutinase_2          TLDQPDSRARQLNAALDYMIND---ASSAVRSRIDSSRLAVMGHSMGGGGTLRLASQRPD      146
TcCutinase_1          TLDQPDSRAEQLNAALNHMINR---ASSTVRSRIDSSRLAVMGHSMGGGGTLRLASQRPD      146
TfCutinase            TLDQPDSRAEQLNAALNHMINR---ASSTVRSRIDSSRLAVMGHSMGGGGTLRLASQRPD      145
Stsα_βHydrolase       TLDQPDSRGRQLLSALDYLTQR-----SSVRTRVDATRLGVMGHSMGGGGSLEAAKSRTS      146
PsLipase              -----AGTGREMLACLDYLVRENDTPYGTYSGKLNTGRVGTSGHSQGGGGSIMAGQD-TR      140
MmaLipaseT            RLDQPDSRGRQLQAALDHLRTN-----SVVRNRIDPNRMAVMGHSMGGGGALSAAANNTS      146
HpDLH                 GFDQPPSRARQINNALDYLVSQNSRSSSPVRGMIDTNRLGVIGWSMGGGGTLRVASE-GR      185
                            .   :   .*  :              ::  * .. * * ****::

                              W185                    D206 I208 N212

IsPETase              LKAAAPQAPWDSST-----NFSSVTVPTLIFACENDSIAPVNSSALPIYDSMSR-NAKQF      229
Sblipase              LKAAVPFAPWHNTA-----NFSGVQVPTLVIACENDTVAPISRHASSFYNSFSSSLAKAY      232
RgDLH                 LRAAAPQAPWAQ-E-----SFSSVTVPTLIVSCENDSIAPNSSHSFPFYNQMTR-NKKAN      226
RdPETase              LKAILPLTPWNLST-----NFSGVQVPTLIVGADGDAVAPVASHARPFYASLPSTVRKAY      226
AqPET_hydrolase       LKASYPMTPWNISS-----NFSTVRVPTMIIGADGDTVAPVGTHARPFYASLPSSTFKAY      221
MsPET_hydrolase       LQAAVPIAPWNTDK-----TWGDVRVPTLIVGGESDSVAPVSSHAVPFYNSIPAASEKAY      240
MesCutinase           LQAAIPLAPWNTTK-----SWSSVRVPTLIIGGEYDTVAPVSSHSEPFYSSIPAASEKAY      240
MgCutinase            LQAAVPLAPWNLDK-----TWSGLRVPTLIVGGETDTIAPVSSHSEPFYNSIPASSEKAY      240
Mcα_βHydrolase        LQAAVPLAPWNLTK-----NWSNLRVPTLIIGGESDTVAPVSSHSEPFYNSIPASAEKAY      218
MmLipaseT             LQAAVPLAPWNLTK-----NWSNLRVPTLIIGGESDSVASVATHSEPFYNSIPASAEKAY      237
MaCutinase            LQAAVPLAPWNLDK-----TWNTLQVPTLIIGGENDSVAPVATHSIPFYNSIPASAEKAY      241
Meα_βHydrolase        LQAAVPLAPWNLDK-----SWSELRVPTLIIGGESDSVASVSSHSIPFYNSIPASAEKAY      239
MaLipaseT             LQAAVPLAPWNLDK-----TWSDVRVPTLIIGGESDSVAPVASHSIPFYNSIPASSEKAY      237
McCutinase            LQAAVPLAPWNLDK-----TWSDVRVPTLIIGGESDSIAPVSSHSEPFYSSIPASSEKAY      240
MefCutinase           LQAAVPLAPWNLDK-----TWSDVRVPTLIIGGESDSIAPVSSHSEPFYNSIPASSEKAY      240
DmLipaseT             LKAAIPLTPWNTDK-----SWPELTTPTLIFGAQNDSVAPVSSHAIPFYTSLASTLPKAY      255
CcLipase              LQAAIPMTPWNTDK-----TWPEVQTPTLIIGAQNDSVASVTTHAEPFYENLPGSLDKAY      265
PmDLH                 LQAAIPLTGWNLRK-----NFSSVRVPTLVVGAENDSVAPVSSHSEPFYASLPSTLDKAY      236
AaPET_hydrolase       LQAAIPLAPWNTDK-----TWNTDQVPTFIIGGESDSVAPVSSHAEPFYTSLTSAPDKAY      228
SsLipase              LQAAIPLTPWNLDK-----SWPEVTTPTLIVGADGDSIAPVSSHAEPFYGSLRSSLDRAY      209
Ssα_βHydrolase        LQAAIPLTPWHTDK-----TWPGVQTPTLVIGAENDSTAPVSSHSEPFYTSMPSTMDKAY      227
Asα_βHydrolase        LQAAIPLAPYHTTK-----NWSTDTVPTLIIGAENDSVAPVSSHAEPFYTSLPASSDKAY      232
AmLipaseT             LQAAIPLAPWHTTK-----NWSSTRVPTMIIGAESDSTAPVSSHSEPFYTSMTSAPDKAY      225
AoCutinase            LQAAIPMTPWHGTK-----SWTTDRVPTMIIGAENDTVAPVSSHAEPFYTSLTAAPDKAY      228
Ksα_βHydrolase        LKAIIPQAPWYSGF----NSFDRITTPTMIIACESDAIAPVGQHASPFYNDIPNSTAKAF      250
Kaα_βHydrolase        LKAAIPQAPWYSGF----NSFNRITTPTLIIACELDVVAPVGQHASPFYNRIPSSTAKAF      251
AxCutinase            LSAAVPLAPWNSGS----NSFNQIAVPTMIMACENDSTANVSSHASPFYNTIPTSTDKAF      247
OaLipase              LSAAIPIAPWNQGG----NRFDQIETPTLVIACENDVVASVNSHASPFYNRIPSTTDKAY      247
AeDLH                 IQAAIGLAPWNTSS----LGFRSIETPTLIFACERDSIAPVRSHASPFYNAIPSSTDKAY      242
HsCutinase            IKAAIPLAPWDTSN----FPSRQVAAPTLIFACESDTVARVGSHASPFYNSISGSIDKAF      249
MdCutinase            LKAAIPLAPWDSSS----TQFRSITAPTLIFACENDAIAPVRSHADPFYNAIPSSTDKAF      188
PyLipaseT             IKAAIPLAPWDTTS----F--RSNRTPTLIFACGSDSIAPVRTHASPFYNQIPTSTPKIF      240
TaCutinase_est2       LKAAIPLTPWHLNK-----SWRDITVPTLIIGAEYDTIASVTLHSKPFYNSIPSPTDKAY      217
TcCutinase_2          LKAAIPLTPWHLNK-----NWSSVRVPTLIIGADLDTIAPVLTHARPFYNSLPTSISKAY      201
TcCutinase_1          LKAAIPLTPWHLNK-----NWSSVTVPTLIIGADLDTIAPVATHAKPFYNSLPSSISKAY      201
TfCutinase            LKAAIPLTPWHLNK-----NWSSVTVPTLIIGADLDTIAPVATHAKPFYNSLPSSISKAY      200
Stsα_βHydrolase       LKAAIPLTGWNTDK-----TWPELRTPTLVVGADGDTVAPVATHSKPFYESLPGSLDKAY      201
PsLipase              VRTTAPIQPYTLGLGHDSASQRRQQGPMFLMSGGGDTIAFPYLNAQPVYRRANV--PVFW      198
MmaLipaseT            LEAAIPLQGWHTRK-----NWSSVRTPTLVVGAQLDTIAPVSSHSEAFYNSLPSDLDKAY      201
HpDLH                 IKAAIPLAPWDTTS----YYASRSQAPTLIFACESDVIAPVLQHASPFYNSLPSSIDKAF      241
                      : :      :                * ::..   *  *     :  .*

                          H237 S238

IsPETase              LEINGGSHSCANSGNS---NQALIGKKGVAWMKRFMDNDTRYSTFACENPNSTRVSDF--      284
Sblipase              LEINNGSHTCANTGNS---NQALIGKYGVAWIKRFVDNDTRYSPFLCGAPHQADLRSSRL      289
RgDLH                 LVINGGSHSCANSGNS---DAGLIGKYGVAWMKRFMDDDTRYSKFLCGAEHQADLSKRAV      283
RdPETase              GELNMASHSTPTSGVN-----TPIGRYGVTWMKRFVDGDTRYSTFLCGAEHQAYATATVF      281
AqPET_hydrolase       GELNGATHFAPNT-TS-----TPIGRYAVAWMKRFVDGDTRYSPFLCGAQHTSYATGLVF      275
MsPET_hydrolase       LELNNASHFFPQT-TN-----TPLAKQMVSWLKRFVDNDTRYDQFLCPGPSGV-----AI      289
MesCutinase           LELNGESHFFPQS-AD-----ALVGKQMVSWLKRFVDNDTRYEPFLCPGPSGL-----DV      289
MgCutinase            LELNGAGHFFPQS-VN-----TPTAKQMVSWLKRFVDNDTRYEQFLCPGPTGL-----AI      289
Mcα_βHydrolase        LELNNASHFFPQS-VN-----TPTAKQMVSWLKRFVDDDTRYEQFLCPGPSGL-----AI      267
MmLipaseT             LELNNEGHFFPNT-VN-----TPTAKQMVAWLKRFVDDDTRYDQFLCPGPSGL-----AV      286
MaCutinase            LELNGASHFFPQT-VN-----TPTARQAVAWLKRFVDNDTRYEQFLCPGPSGS-----AI      290
Meα_βHydrolase        LELNGASHFFPQT-TN-----TPTARQMVAWLKRFVDDDTRYEQFLCPGPTGS-----AI      288
MaLipaseT             LELNGASHFFPQT-VN-----TPTAKQTVAWLKRFVDDDTRYEQFLCPGPTGS-----AI      286
McCutinase            LELNGASHFFPQT-VN-----TPTAKQMVAWLKRFVDNDTRYEQFLCPGPSGF-----EI      289
MefCutinase           LELNGASHFFPQT-VN-----TPTARQAVAWLKRFVDDDTRYEQFLCPGPSGS-----AV      289
DmLipaseT             LELRGASHGAPTS-TN-----TTIAKYAVAWLKRFEDADRRYDPFLCPTPAVS----TLL      305
CcLipase              LELRGASHFAPNT-SN-----TTIARQSIAWLKRFVDDDERYAQFLCPPPSGT-----AI      314
```

```
PmDLH                 LELDNASHFAPNV-SN-----TTIAKYSISWLKLFIDNDTRYAQFLCPAPDDR-----EI     285
AaPET_hydrolase       LELNNASHFFPNT-PN-----TTQAKYMISWLKRFVDNDTRYEQFLCPGPSKS----TLV     278
SsLipase              LELNGASHFTPNS-SN-----TTIAKYSVSWLKRFIDNDTRYEQFLCPLPSPS----LTI     259
Ssα_βHydrolase        LELNGASHSAPSS-SN-----TTIAKYSIAWLKRFIDNDTRYEQFLCPAPARG----TLI     277
Asα_βHydrolase        LELNNASHSAPTS-TN-----VTIAKYSISWLKRFIDDDTRYEQFLCPAPSGS-----TI     281
AmLipaseT             LELNNAGHSAPTS-AN-----TTVAKYSISWLKRFIDNDTRYDQFLCPAPSPS----SLI     275
AoCutinase            LELNNASHSAPTS-AN-----TTVAQYSISWLKRFIDNDTRYDQFLCPAPSPS----TLI     278
Ksα_βHydrolase        LEINGGSHYCANSGYS---DEDILGKYGISWMKRFMDNDTRYSQFLCGPNHES---DRSI     304
Kaα_βHydrolase        LEINGGDHFCANSGYP---NEDILGKYGVSWMKRFIDGDRRYDQFLCGPNHES---DRSI     305
AxCutinase            MEISGGQHNCANGSNS---NNALLATYGVSWMKRFLDKDHRYNQFLCGPNHES---NSRI     301
OaLipase              LEINGGSHFCANDGGS---IGGLLGKYGVSWMKRFIDNDLRYDAFLCGPDHAA---NRSV     301
AeDLH                 FEISGGNHYCANGSNR---YDALLGKYGVAWMKLHLDQDQRYAPFLCGPNHER---DRQI     296
HsCutinase            VELDNGNHYCANGGNDRGRYDAVLGRMGVSWMKRFLDEDTRYSQFLCGPNHTS---DRQI     306
MdCutinase            FELNGGSHYCANGGNS---YNDELSRLGVSWMKLHLDKDQRYNQFLCGPNHES---DWNI     242
PyLipaseT             IEINRGNHYCANGGNS---NNATLGRLGVAWMKRFLDEDRRYSQFLCGPRHTS---DRNI     294
TaCutinase_est2       LELDGASHFAPNI-TN-----KTIGMYSVAWLKRFVDEDTRYTQFLCPGPRTGLL--SDV     269
TcCutinase_2          LELDGATHFAPNI-PN-----KIIGKYSVAWLKRFVDNDTRYTQFLCPGPRDGLF--GEV     253
TcCutinase_1          LELDGATHFAPNI-PN-----KIIGKYSVAWLKRFVDNDTRYTQFLCPGPRDGLF--GEV     253
TfCutinase            LELDGATHFAPNI-PN-----KIIGKYSVAWLKRFVDNDTRYTQFLCPGPRDGLF--GEV     252
Stsα_βHydrolase       LELRGASHFTPNT-SD-----TTIAKYSISWLKRFIDSDTRYEQFLCPIPRPS----LTI     251
PsLipase              GERRYVSHFEPVGSG------GAYRGPSTAWFRFQLMDDQDARATFYGAQCSLCTSLLWS     252
MmaLipaseT            MELRGASHLVSNT-PD-----TTTAKYSIAWLKRFVDDDLRYEQFLCPAPDDF-----AI     250
HpDLH                 VEINGGSHYCGNGGSI---YNDVLSRFGVSWMKLHLDEDSRYKQFLCGPNHTS---DSQI     295
                             *                     :*::     *

IsPETase              --RTANCS---------290
Sblipase              SEYRESCPY--------298
RgDLH                 EAYKENCPY--------292
RdPETase              DRYSQNCPY--------290
AqPET_hydrolase       DRYNQNCPY--------284
MsPET_hydrolase       QEYRNTCPHA-------299
MesCutinase           EEYRNTCPSS-------299
MgCutinase            EEYRDTCPHS-------299
Mcα_βHydrolase        EEYRNTCPH--------276
MmLipaseT             EEYRNTCPH--------295
MaCutinase            EEYRNTCPSA-------300
Meα_βHydrolase        EEYRNTCPSS-------298
MaLipaseT             EEYRNTCPSS-------296
McCutinase            QEYRNTCPSY-------299
MefCutinase           QEYRNTCPSA-------299
DmLipaseT             SDARSTCPFN-------315
CcLipase              SEYRSTCPY--------323
PmDLH                 SEYRETCPYSP------296
AaPET_hydrolase       EEFRSTCPNG-------288
SsLipase              EEYRGNCPHTS------270
Ssα_βHydrolase        EEYRDTCPHTA------288
Asα_βHydrolase        QEYRDTCPHS-------291
AmLipaseT             EEYRATCPHS-------285
AoCutinase            QEYRDTCPHS-------288
Ksα_βHydrolase        SEYRDTCNY--------313
Kaα_βHydrolase        SDYRETCNY--------314
AxCutinase            SEFRDTCDFT-------311
OaLipase              SEYRDTCNY--------310
AeDLH                 SEHRSTCPF--------305
HsCutinase            SEYRGNCPY--------315
MdCutinase            SEYRGTCPY--------251
PyLipaseT             SESRDTCNF--------303
TaCutinase_est2       EEYRSTCPF--------278
TcCutinase_2          EEYRSTCPFALE-----265
TcCutinase_1          EEYRSTCPFALE-----265
TfCutinase            EEYRSTCPF--------261
Stsα_βHydrolase       AEYRGTCPHTS------262
PsLipase              VERRGL-----------258
MmaLipaseT            SEYRSTCPFLEHHHHHH267
HpDLH                 SDYRGNCPYLEHHHHHH312
```

Figure S1. Multiple sequence alignment of *Is*PETase homologous enzymes. The arrows indicate the positions of key residues. The catalytic triad, the residues flanking S160 and the residues mutated in the present study are highlighted green, yellow and magenta, respectively.
